# Marine Metagenomics: New Tools for the Study and Exploitation of Marine Microbial Metabolism

**DOI:** 10.3390/md8030608

**Published:** 2010-03-15

**Authors:** Jonathan Kennedy, Burkhardt Flemer, Stephen A. Jackson, David P. H. Lejon, John P. Morrissey, Fergal O’Gara, Alan D. W. Dobson

**Affiliations:** 1 Marine Biotechnology Centre, Environmental Research Institute, University College Cork, Cork, Ireland; E-Mails: jonathan.kennedy@ucc.ie (J.K.); bflemer@web.de (B.F.); s.a.jackson@student.ucc.ie (S.A.J.); d.lejon@ucc.ie (D.P.H.L.); j.morrissey@ucc.ie (J.P.M.); f.ogara@ucc.ie (F.O.); 2 Department of Microbiology, University College Cork, Cork, Ireland; 3 BIOMERIT Research Centre, University College Cork, Cork, Ireland

**Keywords:** metagenome, marine, microorganism, enzyme

## Abstract

The marine environment is extremely diverse, with huge variations in pressure and temperature. Nevertheless, life, especially microbial life, thrives throughout the marine biosphere and microbes have adapted to all the divergent environments present. Large scale DNA sequence based approaches have recently been used to investigate the marine environment and these studies have revealed that the oceans harbor unprecedented microbial diversity. Novel gene families with representatives only within such metagenomic datasets represent a large proportion of the ocean metagenome. The presence of so many new gene families from these uncultured and highly diverse microbial populations represents a challenge for the understanding of and exploitation of the biology and biochemistry of the ocean environment. The application of new metagenomic and single cell genomics tools offers new ways to explore the complete metabolic diversity of the marine biome.

## 1. Introduction

The marine environment is by far the largest habitat on Earth, with oceans covering ~70% of the earth’s surface. Within this environment, habitats range from tropical sunlit surface waters to ocean trenches 11,000 m deep with pressures exceeding 100 MPa. While salinity is fairly uniform at 3.5%, temperatures range from sea ice in the polar regions to temperature in excess of 100 °C at deep sea hydrothermal vents. Microorganisms are able to survive and grow throughout this environment, either surviving as free-living organisms or in association with other organisms. The marine environment is dominated by microorganisms from the three domains of life, namely Archaea, Bacteria and Eukarya.

Microorganisms are believed to be responsible for up to 98% of marine primary productivity [1], playing key roles in marine food webs and in carbon and energy cycles. Microorganisms dominate the oceans in terms of total biomass and metabolic activity; with pelagic photosynthetic microorganisms producing the majority of organic matter present within the oceans, while at the other end of the food web, sediment microorganisms degrade dead marine macro organisms and marine snow. The different environmental challenges within the marine environment have led to different microbes adopting different survival and growth strategies. For example, the photosynthetic pelagic bacterium *Prochlorococcus marinus* MED4 has adopted a minimalist approach, with conditions within its environmental niche varying little, the bacterium has reduced its genome size to 1.66 Mbp to gain a competitive advantage [2]; while *Pelagibacter ubique* from the SAR11 clade of marine alphaproteobacteria has no transposons, extrachromosomal elements, pseudogenes or introns and constitutes the smallest genome known for a free-living microorganism, encoding the smallest number of predicted open reading frames [3]. Adaptation to the challenges of the deep sea, where nutrient sources, e.g., from marine detritus, can be unpredictable; has led to greater adaptability in both signaling pathways involved in the detection of nutrients and greater metabolic capabilities in utilizing them. Likewise, microorganisms from the extremes within the marine environment have adapted to the extremes of temperature and pressure, resulting in novel biochemistry which is able to operate at high or low temperatures and pressures, perhaps uniquely suited for many industrial processes.

Life in the deep-sea can also occur entirely independently of sunlight driven photosynthesis. At hydrothermal vents, for example, chemolithoautotrophic bacteria are the primary producers for these unusual ecosystems, with archaea isolated from these environments able to grow at 121 °C. Photosynthetic bacteria entirely dependent on the geothermal radiation from these vents have also been isolated [4]. Cold seeps, in which hydrocarbon rich fluids seep from the ocean floor, are also hotspots of diversity, in which the primary producers are chemosynthetic microorganisms [5]. These discoveries at the extremes of ocean life have led to a significant extension of the known biosphere.

Thus, the ability of microbial life to thrive throughout the entire marine environment, from deep ocean vents to surface sea ice, is entirely due to the diverse and adaptable biochemistry encoded within their genetic resources. In this review, we highlight recent discoveries in the study of enzymes from marine microbial sources, and advances in the technologies available to study the vast genetic resources of marine microbiota.

## 2. Molecular Approaches to Study Marine Microbial Biodiversity

It is now widely accepted that <1% of microbes can be isolated using traditional culturing techniques, leaving the vast majority of microorganisms and their biochemical pathways inaccessible. The emergence of culture-independent and metagenomic techniques has however provided us with the tools to determine the full extent of the uncultured microbial diversity and allowed access to the biochemical pathways within these uncultured microorganisms. Metagenomics is defined as the study of the pooled genetic complement of a given environmental sample, and analyses can be either sequence driven or function driven [6]. To date, sequence-based metagenomic analyses of marine microbiota have attempted to describe; ‘who is there?’; ‘what are they doing?’; ‘who is doing what?’ and ‘what evolutionary processes determine these parameters?’ Recent conceptual and technological advances have allowed the increased use of sequence guided metagenomic investigations of the marine environment. Recent advances in high-throughput sequencing technology and lower cost sequencing technologies have made random shotgun sequencing of environmental DNA economically feasible. The majority of metagenomic investigations to date have employed whole metagenome shotgun sequencing approaches for the cloning and sequencing of microbial DNA from marine environments. This involves the generation of small-insert DNA clone libraries, and their subsequent analysis using Sanger dideoxy sequencing; generating sequences that can then be used to query databases, whereby phylogeny can be inferred or putative functional genes identified. Where putative protein encoding sequences are detected proximal to phylogenetic marker genes on a single cloned fragment, function can be linked to taxonomy. This approach can give read lengths ranging from 600 to 900 bp in length, which can be extended through the entire fosmid clone. The contigs generated using this approach give a fuller context for any given gene detected, thus giving a greater probability of reconstructing the metabolic pathways of individual members within a particular marine microbial consortium.

454 pyrosequencing, involving a novel sequencing-by-synthesis technology, is also now gaining popularity as a cost effective methodology that can be employed for metagenomic analysis of microbial communities. While the benefits of the technology both from a sequence data generation and cost standpoint (25 to 40 Mb of DNA sequence per run at an accuracy of ~98% [7]) are obvious, the limitations of lower read lengths of between 200 to 300 bp can be problematic [8]. Read lengths achievable using pyrosequencing are currently around 450 bp, and this is likely to continue to improve, which should improve the amount of useful data generated from pyrosequencing of metagenomic samples.

The shorter contigs generated with this method, often do not enable the assignment of function, and reconstruction of the metabolic pathways present in individual microbes is only possible for the simplest of consortia. However notwithstanding this, these random shotgun sequencing and 454 pyrosequencing approaches require no prior sequence knowledge for data generation, and thus there is a good possibility that novel gene sequences can be discovered.

The assignment of taxonomic status to these sequences requires the use of recently developed software such as Metagenomic And rDNA Taxonomy Assigner (MARTA), or Genomic Signature based Taxonomic Classifier GSTaxClassifier. MARTA, which is available at http://bergelson.uchicago.edu/software/marta, allows the classification of taxonomic status using percent-identity and thresholds; employing DNA sequence data from 5,000 typed bacterial species, which allows results generated with MARTA to be compared to those from the phylogenetic algorithm CARMA and the ribosomal database project RDP-II Classifier [9]. GSTaxClassifier, which is available at http://helix2.biotech.ufl.edu:26878/metagenomics/, is a program that allows the evaluation of genomic signatures between metagenomic query sequences and reference genome databases [10]. The assignment of function typically requires profile-to-sequence searches, whereby predicted protein sequences are compared to sequence alignment profiles from the protein families PFAM [11], COGs [12] and TIGRFAM [13], using RPS-BLAST [14]. PFAMs facilitates both the identification and annotation of protein domains, TIGRFAM includes models for both domain and full-length proteins, while COGs also allows the annotation of full-length proteins. InterPro, an integrated protein database, is also widely used for the classification and annotation of proteins and is hosted at http://www.ebi.ac.uk/interpro/. Thus, a number of bioinformatic tools now exist for data mining of metagenomic sequences, based not only on primary sequence homology but also on predicted protein structures. It can therefore be anticipated in the near future that searches of sequence-based metagenome databases in combination with bioinformatic tools, some of which will be discussed later in this review, will be equally as powerful an approach as functional-based methods in the mining of novel genes encoding enzymes from marine microbes.

### 2.1. Who is there?

Diversity and the abundance of free-living marine microorganisms have been described using 16S rRNA based analyses and total metagenomic analyses from a variety of different marine environments such as in ocean surface waters [15,16], mesopelagic waters [17], the deep sea [1,18,19], water columns [20] and sea subfloor sediments [21]. The microbial diversity of marine invertebrate-associated organisms has also been investigated including marine corals [22,23] and marine sponges [24–26]. A brief summary of the results obtained will now be described.

In studies on surface waters of the Sargasso Sea [16], different phylogenetic biomarker genes were used to elucidate the composition of the bacterial community, resulting in the identification of nine major bacterial phyla (*Proteobacteria*, *Actinobacteria*, *Cyanobacteria*, *Firmicutes*, *Bacteroidetes*, *Chloroflexi*, *Spirochaetes*, *Fusobacteria* and *Deinococcus-Thermus*) and two archaeal phyla (*Crenarchaeota* and *Euryarchaeota*). Over 1800 bacterial species, including 148 new phylotypes, were detected, with the vast majority of all sequences clustering within the α- and γ-divisions of the phylum *Proteobacteria*.

In studies on mesopelagic waters involving analyses of the Bermuda Atlantic Time-Series (BATS), the *SAR202* clade of Green Non Sulphur (GNS) bacteria were shown to be prevalent at the interface of the photic and euphotic zones and rare in the immediate sub-surface [17]. Studies on the deep ocean, involving analysis of a metagenomic fosmid library created from seawater sampled at a depth of 3,000 m in the Ionian Sea, revealed sequences related to *Acidobacteria*, *Chloroflexi*, *Planctomycetes* and *α-* and *γ-Proteobacteria,* while 28 archaeal sequences related to *Crenarchaeota symbosium* were obtained [18]. Other work investigating a 16S clone library from a depth of 500 m in the Antarctic Ocean revealed a novel group of *δ-Proteobacteria* with low homology to previously known organisms [27].

Studies on the microbial diversity in depth specific profiles of water columns have also been undertaken, such as the comparative analysis of datasets from the Greenland Sea, the Ionian Sea and the Sargasso Sea surface water (5 m) [19]. This study identified that—despite differences between sample sites: Sargasso Sea (5 m), Greenland Sea 50m (cold) and Ionian Sea 50 m (warm), and differences in the methodologies used—clear vertically stratified community differences were evident and certain similarities between samples were obvious, in particular the widespread presence of DNA sequences that shared homology with genes from *Pelagibacter ubique.* Studies on the microbial community structure from sub-seafloor sediments in the Peru Margin have reported a consortium dominated by *Crenarchaeota,* with *Euryarchaeota* and *Chloroflexi* also abundant [21].

Microbial communities form specific intimate and incidental associations with many marine invertebrates and the microbiota associated with several sponge species in particular have been widely investigated [24,25,28–31]; particularly since microbial endosymbionts of marine sponges are thought to be a rich source of marine natural products [32]. A good recent example is the study from the Wagner group, which employed 16S rRNA tag pyrosequencing to investigate the bacterial diversity associated with three sponge species and the surrounding seawater. Over 250,000 sequences were generated, which aligned to 23 bacterial phyla, demonstrating a much higher diversity than had previously been reported [26]. Metagenomic analyses have also been reported from corals [22,23,33], with fungal sequences accounting for 38% of the classified sequences, and the remainder of the identified sequences aligning to bacteria (7%), archaea (1%), eukaryotic viruses (2%) and phage (3%). The bacterial sequences identified were most similar to *Proteobacteria*, *Firmicutes*, *Cyanobacteria* and *Actinobacteria*.

### 2.2. Resources for marine metagenomic analysis

With the advent of the “omics” era, more and more metagenomic and metatranscriptomic datasets are being generated from marine environments. Analysis resources and tools have been developed in an attempt to maximize the analysis of data that can be obtained from these vast datasets. For example, for marine-derived data, a dedicated web based resource, the Community CyberInfrastructure for Advanced Marine Microbial Ecology Research and Analysis (CAMERA http://camera.calit2.net/), has been developed [34]. The CAMERA database hosts marine metagenomic datasets and completed marine microbial genome sequences as well as sample metadata such as sample site GPS location, depth, temperature, salinity, *etc*. Additional tools available for metagenome analysis include: the Integrated Microbial Genome/Microbiome (IMG/M) http://img.jgi.doe.gov/cgi-bin/m/main.cgi [35], which provides tools for analyzing the functional capability of microbial communities based on their metagenome sequence by allowing comparative analysis of genome and metagenome sequences available on the JGI-Integrated Microbial Genomes system; MetaBioME [36], which is a resource specifically aimed at novel enzyme discovery from metagenomic data; and Orphelia, which is a tool for discovering ORFs in short metagenomic sequences of unknown phylogeny [37]. Recently, the Metagenomics RAST server hosted at http://metagenomics.nmpdr.org/ has been launched, which provides a fully-automated service for annotating metagenome samples. Not only does it provide annotation of sequence fragments, but it also provides a means for comparing phylogenetic classifications and metabolic reconstructions of metagenomes [38]. Other resources include JANE hosted at http://jane.bioapps.biozentrum.uni-wuerzburg.de, which facilitates the mapping of prokaryotic Expressed Sequence Tags (ESTs) or variable sequence reads from DNA sequence and transcriptomic data to related template genomes [39]. WebCARMA hosted at http://webcarma.cebitec.uni-bielefeld.de has recently been developed to facilitate the functional and taxonomic classification of unassembled metagenomic reads as short as 35 bp [40]. MEGAN, a standalone downloadable computer programme for the analysis of metagenomic data [41], has also recently been extended to allow the comparative analysis of multiple datasets, specifically allowing visual and statistical comparison of metagenomes [42]; and can be obtained from http://www-ab.informatik.uni-tuebingen.de/software/megan. As the major drawback to metagenomic data analysis is the lack of reference sequences and genomes, the increased data and tools available should gradually minimize this problem. Also, the inclusion of comprehensive sampling metadata will assist in contextualizing sequence data for improved inferences. Thus, the rapidly developing field of sequence based metagenomic analysis of marine microbiota promises much for the future of marine biotechnology.

## 3. Functional Metagenomic Based Approaches

While sequence based metagenomic approaches rely on comparing sequence data, which is obtained with sequences deposited in databases, functional metagenomics focuses on screening DNA library clones directly for a phenotype, *i.e.,* the genes are recognizable by their function rather than by their sequence. The power of such an approach is that it does not require the genes of interest to be recognizable by sequence analysis, ensuring that this approach has the potential to directly identify entirely new classes of genes for both known and indeed novel functions [6,43]. Another advantage of such an approach is that while sequence based approaches can result in the incorrect annotation of sequences with weak similarities to biochemically characterized gene products or of sequences similar to gene products with multiple functions, the results from a functional metagenomics approach are unambiguous.

**Figure 1:**
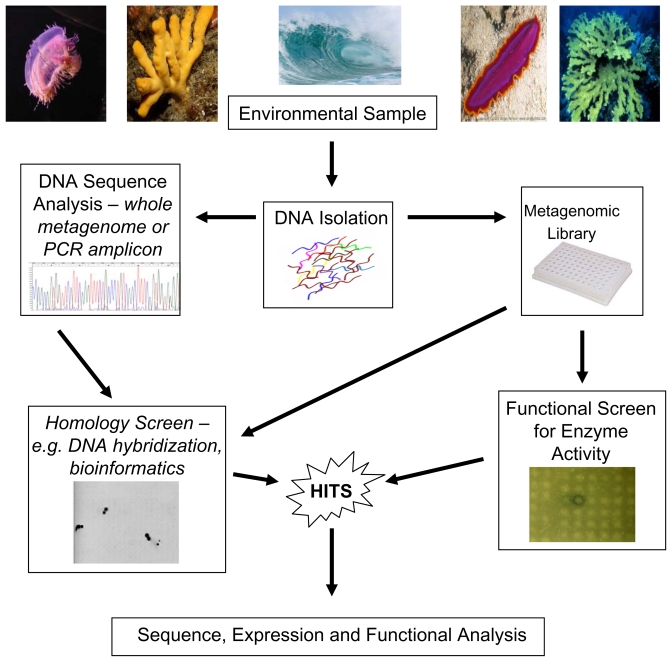
Enzyme discovery from metagenomes: functional and sequence-based approaches.

The basic strategy for functional metagenomics is outlined in Figure 1. An environmental sample, e.g., seawater or marine invertebrate is collected and then total community DNA is extracted from the sample. The isolated DNA is then used to generate a metagenomic library using a suitable cloning vector. This library is then transferred to a suitable host strain, usually *Escherichia coli*, and individual clones can then be screened for the presence of enzymatic or other bioactivities encoded by the environmental DNA fragment. When coupled with a robust and high-throughput screen, this method is an extremely effective way of isolating novel enzymes from otherwise inaccessible microbes. Examples of enzymes isolated from marine sources using a functional metagenomics approach are outlined in Table 1; these include esterases, lipases, chitinases, amylases and amidases. While the functional based approach has been successfully applied to terrestrial environments, especially soil, with the discovery of new genes for antibiotics, antibiotic resistance and industrial enzymes [44–46], the full potential of marine functional metagenomics has to date remained largely unrealized. It is estimated that about 1 × 10^6^ bacteria are present in 1 mL of seawater, with far greater concentrations in marine snow [47–49]. However, only about 0.0001–0.1% are believed to be culturable [50], making a culture independent approach to harvest the metabolic potential of these organisms even more attractive. In addition, data from the Global Ocean Sampling (GOS) expedition indicates that despite current large-scale sequencing efforts the rate of discovery of new protein families from the marine environment is linear, implying that marine microorganisms will continue to be a source of novel enzymes in the foreseeable future [51]. As many of these gene products are entirely novel, their activity cannot be inferred from comparison to known protein databases, thus a functional metagenomics approach has the ability to identify novel genes on the basis of phenotypes which lend themselves to high throughput screens.

As can be seen in Table 1, functional metagenomic based approaches have led to the discovery of many new enzymes from marine sources. However, despite the undoubted promise of functional metagenomics for the discovery of new enzymes, the approach is limited to some extent by the ability of metagenomic clones to produce active enzymes. Many functional metagenomic approaches rely on the use of *E. coli* as a host for the expression of metagenome encoded proteins. While quite a large number of genes derived from *Enterobacteriaceae* will be readily expressed in the most common *E. coli* host, many genes from more distantly related organisms may not be expressed due to the promoter regions of these genes not being recognized by the *E. coli* transcriptional machinery or be expressed at low levels due to differences in codon usage, for example. Even where transcription and translation of foreign genes results in efficient protein expression, additional problems can arise when proteins need to be post-translationally modified or exported for activity. For these reasons, the availability of suitable heterologous expression hosts remains a barrier to extracting the maximum information from functional metagenomic analyses [6].

On the other hand, due to advanced screening methodologies and the use of robotic instrumentation, it is now possible to screen large clone libraries for functional activities in a high throughput fashion, in relatively short timescales [52]. The development of new hosts for heterologous expression will also contribute to advances in function-based metagenomic approaches. For example, *Streptomyces lividans*, *Pseudomonas putida* and *Rhizobium leguminosarum* have all been assessed for their potential as expression hosts for metagenomic libraries [53–55]. Other approaches have looked at ways of increasing the levels of functional expression of foreign genes in *E. coli.* Issues such as restricted expression due to codon usage have been addressed by supplementing *E. coli* with plasmids containing additional tRNA genes [56]. Inadequate folding of target proteins can be addressed through co-expression of chaperone proteins [56]. Indeed, the expression of the chaperonin 60 gene and the cochaperonin 10 gene from the psychrophilic bacterium *Oleispira antarctica* RB8T can effectively lower the minimum temperature for growth of *E. coli*, allowing the functional expression of cold-adapted proteins [57].

Coupling classical functional screens of metagenomic libraries with innovative approaches such as substrate-induced gene expression screening (SIGEX), pre-amplification inverse-PCR (PAI-PCR) and metagenomic DNA shuffling can also further improve function-based metagenomics. SIGEX was initially developed to detect metagenomic clones that express desired catabolic genes induced by the presence of suitable substrates using green fluorescent protein (GFP) as a reporter gene [58]. Constitutively expressing clones are first removed by fluoresence activated cell sorting (FACS), then the expression of desired genes is induced by the addition of substrates and positive clones are again sorted by FACS, separated on agar plates and characterized [59]. With SIGEX, genes possibly involved in benzoate and catechol degradation have been discovered in a groundwater metagenomic library [58]. Despite the limitations of SIGEX, which can only detect genes that are correctly induced in a heterologous host, the method is extremely high throughput since large number of clones can be screened in relatively short timescales [60].

Inverse PCR (I-PCR) is a method that can be used for cloning the upstream and downstream flanking regions of known sequences [61], and has been employed in the amplification of known gene families. However, I-PCR has found little utility when applied to metagenomes, presumably due to the potential increased complexity and low copy numbers of target sequences. Pre-amplification of the template DNA, using phi29 DNA polymerase coupled with inverse PCR (PAI-PCR), has, however, been shown to facilitate the isolation of novel genes from small amounts of environmental DNA [61]. Using PAI-PCR, DNA glycosyl hydrolase genes have been identified, with a 10^5^-fold increase in sensitivity compared to I-PCR without pre-amplification [61]. Thus, this approach could also be useful for marine DNA-samples where the amount of extracted DNA is low. Finally, metagenomic DNA shuffling for its part simulates lateral gene transfer and gene rearrangements in microbial genomes. This approach has been used to generate novel lindane degrading genes in terrestrial systems [62]. The advantages of this method are that the diversity of the metagenome can actually be increased, and by applying stringent selection steps enzymes particularly suited to industrial applications can be generated. Thus, such an approach could also be useful to find novel enzyme encoding genes in the marine environment.

## 4. Marine Microbes and Their Potential for Functional Metagenomics

### 4.1. Biotechnological uses of marine enzymes

The potential for novel biochemistry in the marine environment has not been overlooked, especially in the field of specialty and diagnostic enzymes. The luminescent properties of the jellyfish *Aequorea victoria* led to the characterization of the green fluorescent protein (GFP), which now has widespread uses in molecular biology as a reporter protein [63], and has been utilized in studies involving bacteria, fungi, fish and mammalian cells. Similarly, the protein aequorin, also from the *Aequoria* jellyfish, has found use as a biosensor for Ca^2+^ signaling as it emits a blue light in the presence of Ca^2+^ ions [64]. Microbial light emitting systems have also been discovered, the most well known of which is the bioluminescent bacterium *Vibrio fischeri*, which is found within squid light organs. Luciferase, the enzyme responsible for this light emission, has also found widespread use as a reporter system [65].

For research and other purposes, enzymes isolated from microbial sources at the extremes of temperature have proven to be particularly useful. DNA polymerases isolated from thermophilic marine eubacteria (Tth polymerase) and archaea (VENT® polymerase) have proved useful alternatives to Taq DNA polymerase, while thermolabile phosphatases have also found utility in the research laboratory. In the bulk enzyme market, a heat and acid stable α-amylase, Valley Ultra-Thin, discovered from deep sea hydrothermal vent archaea, has been developed by Diversa/Verenium to facilitate the processing of corn into ethanol [66].

### 4.2. Novel enzyme discoveries

The potential of marine microorganisms for the discovery of new enzymes has led to numerous new discoveries in recent years (Table 1). These discoveries have been made from both microorganisms that have been isolated from the marine environment and from metagenomic libraries. The spectrum of marine-derived genes coding for enzymes with unique abilities includes lipases [67], cellulases, proteases, alkane hydroxylase genes [68], esterases with high tolerances for salt and organic solvents [69], and metalloproteases with a high temperature optima [70].

Esterases have potential commercial value for use in industrial biotransformations. Cold active esterases have been isolated from *P. haloplanktis* [71], and from metagenomic libraries, esterases with preferences for high pressure and salt [72] and with high tolerance for organic solvents [69] have been discovered. Lipases have wide ranging applications in the food, detergent, and pharmaceutical industries, and marine microorganisms have proven to be a source of novel lipases with new families of lipase [73,74] and cold active lipases [75] discovered from isolated microorganisms and metagenomes. Cellulases, a class of enzyme under intense study for their role in the generation of biofuels from renewable cellulosic substrates, have been discovered from various marine microorganisms including an alkaline cellulase, stable to pH 12, from a bacteria isolated from a sponge [76], cold active cellulases [77,78] and cellulases from bacteria isolated from a shipworm [79]. A shipworm metagenome project is also currently being pursued by the U.S. Joint Genome Institute to better understand the role of the shipworm microbiota in the degradation of wood. Chitin is also a major marine carbon source and chitinases have been discovered in metagenomic libraries [80] and a thermostable chitinase has been purified from a marine Bacterium [81]. Hydrocarbon cold seeps and oil spills have led to the adaptation of certain marine microbiota to utilize alkanes as carbon sources. Such bacteria and their enzymes have a potential role in bioremediation and oil processing. Alkane hydroxylase genes encoding enzymes with specificity for alkanes of varying lengths have been isolated from deep sea and cold seep metagenomic libraries [68,82].

### 4.3. Phenotypic screens

Robust and sensitive screens are essential for the discovery of novel biocatalysts. As previously mentioned, sequence-based approaches rely on homologies to known protein families; a drawback that does not apply to functional screens [83]. The most commonly reported type of functional screen for the discovery of enzymes from metagenomic libraries is based on colony phenotype changes on agar plates. These assays typically rely on the utilization of a substrate resulting in the appearance of a zone of clearing around positive clones.

**Figure 2:**
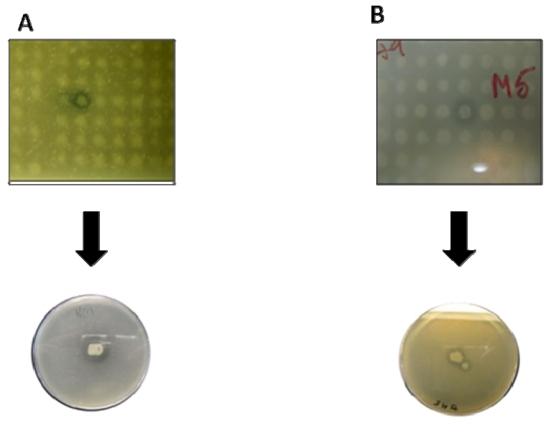
Examples of positive lipase (A) and protease activities (B) from a *Haliclona simulans* metagenomic library.

For example, our group has detected both protease and lipase activity in clones from a *Haliclona simulans* sponge metagenomic library by replicating the library onto Luria-Bertani containing skimmed milk [84], or in the case of lipase activity, supplemented with tributyrin [85]. Clear halos around these colonies indicate either protease or lipase activity in each case (Figure 2). Chromogenic substrate analogs can also be employed to improve the sensitivity of these assays, with positive clones causing a loss or appearance of color. One major advantage of using agar plate assays is that they are highly scalable and with integrated robotics many thousands of clones can be screened per day. A major disadvantage, however, is that agar plate assays often require the active transport of either the substrate or the enzyme to detect activity, and it is widely believed that many potentially novel enzymes are in many cases simply not detected as they fail to come in contact with the substrate. To circumvent this problem, cell extracts have been successfully employed to identify novel genes involved in aromatic compound degradation [46]. Additional screening approaches that can be employed include phenotypic screens where the introduction of a new catabolic pathway into a host strain allows it to subsequently grow on a previously inaccessible substrate. A variation on this approach is the use of an engineered host that lacks a particular activity, and the reintroduction of this activity via a metagenomic clone, thereby providing a straightforward method by which to screen very large numbers of clones in a metagenomic library for novel activities. In addition, the utilization of engineered host strains can allow screens to pass beyond the simple growth / no growth assay based systems. For example, engineered strains in which the induction of a specific pathway is linked to a reporter gene can be used to isolate metagenomic clones that induce particular cellular pathways. This strategy has been used to identify clones affecting signal transduction from a moth gut metagenome [86]. Thus, the use of sensitive biosensor host strains such as these provides much promise for the discovery of genes encoding novel properties from marine metagenomic libraries.

## 5. Novel Approaches

New approaches, which are likely to have a major impact on the discovery of novel enzymes from marine microorganisms that are difficult to culture, include single cell genomics, metatranscriptomics and metaproteomics. While the metagenomics approaches described above are useful for exploiting the biochemistry of microbial communities, they are however unable to access the metabolic capabilities associated with specific microorganisms within the consortia; given that they largely rely more on a “take all” approach. While functional metagenomic based approaches have proven to be successful for the discovery of novel enzymes, it is quite likely that many novel enzymes from rare microbes in complex communities are poorly represented in metagenomic libraries. One solution to this problem is the use of multiple displacement amplification (MDA) for whole genome amplification from single cells, which will allow the study of the entire biochemical potential of single uncultured microbes from complex microbial communities [87,88]. In this approach, microbial cells are first sorted using FACS and collected. Then, MDA is used to amplify the entire genome from the sorted cells. The amplified genomic DNA can then be analyzed to infer phylogeny, from 16S rRNA sequence, and used as a template for anchor-based analysis such as PCR, to isolate known families of genes, or can be directly sequenced to access the full metabolic potential of the microorganism. This approach has great potential for the discovery of novel enzymes from marine microbes, as it affords easier access to rare microbiota and should also enable the matching of phylogenetically characterized amplified genomes to suitable expression hosts, thereby increasing the chances of discovering novel biochemistry.

While the standard metagenomic approach of accessing the entire genetic material associated with an environment may well indicate what genes are present within a marine environment, it does not, however, offer clear evidence regarding which genes are active within this environment. To overcome this particular problem, metatranscriptomic based approaches have been employed to study marine microbial populations, in which only transcriptionally active genes are accessed [89]. Applying this approach to the study of ocean waters has resulted in the detection of genes known to be involved in key aspects of the metabolism of open ocean microbial species, and in addition many unique transcripts, presumably also of biochemical importance have been detected [90,91]. Metatranscriptomic analyses can be used to determine the enzymes in an environment that are actively involved in a particular biochemical pathway [92]. An alternative strategy to accessing the metagenome is to directly analyze the proteins within the marine environment using metaproteomics, for review see [93,94]. This has proven useful in providing new physiological data such as the identification of proteins that are essential for stress and starvation resistance in culturable marine bacteria. A recent terrestrial based approach has used the binding properties of specific proteins to isolate them from a particular environment, for example, bacterial cellulose-binding proteins from sheep rumen extracts were isolated and identified in this way [95]. Similarly, an array of dye-linked substrates has recently been used to identify enzymes with specific activities from microbial extracts and from metagenomic libraries [96]. It is clear that by applying novel screening tools such as these to samples from marine ecosystems that novel and metabolically relevant enzymes can be discovered.

## Figures and Tables

**Table 1:** Marine enzymes discovered from Microbial and Metagenomic sources.

Activity	Source	Habitat	Reference

Esterase	Metagenome	Deep-sea sediment	[97]
	Metagenome	Deep-sea basin	[72]
	Metagenome	Surface seawater	[69]
	Metagenome	Arctic sediment	[98]
	*Vibrio* sp.	Sea Hare Eggs	[99]
	*Pseudoalteromonas haloplanktis*	Antarctic Seawater	[71]

Lipase	Metagenome	Tidal Flat	[74]
	Metagenome	Deep Sea sediment	[75]
	Metagenome	Baltic Sea sediment	[67]
	*Pseudoalteromonas haloplanktis* TAC125	Antarctic Seawater	[73]
	*Aureobasidium pullulans* HN2.3	Sea saltern	[100]

Cellulase	*Pseudoalteromonas* sp. DY3	Deep-sea sediment	[77]
	*Pseudoalteromonas haloplanktis*	Antarctic Seawater	[78]
	*Teredinibacter turnerae* T7902T	Shipworm	[79]
	*Marinobacter* sp. MSI032.	Marine sponge	[76]

Chitinase	Metagenome	Estuary	[80]
	*Arthrobacter* sp. TAD20	Antarctic ice	[101]
	*Rhodothermus marinus*	Marine hot spring	[81]

Amidase	Metagenome	Marine sediments/sludges	[102]

Amylase	*Aureobasidium pullulans* N13d	Deep-sea sediment	[103]
	Metagenome	Deep sea hydrothermal vent	[66]

Phytase	*Kodomaea ohmeri* BG3	Fish gut	[104]

Protease	*Pseudomonas* strain DYA	Deep-sea sediment	[105]
	Marine bacterium	Antarctic Seawater	[106]
	*Aerpyrum pernix* K1	Coastal solfataric vent	[107]

Alkane hydroxylase	Metagenome	Hydrocarbon seep	[82]
	Metagenome	Deep sea sediment	[68]

Xylanase	*Pseudoalteromonas haloplanktis*	Antarctic Seawater	[108]
